# The Living Lab in Ageing and Long-Term Care: A Sustainable Model for Translational Research Improving Quality of Life, Quality of Care and Quality of Work

**DOI:** 10.1007/s12603-019-1288-5

**Published:** 2019-10-25

**Authors:** Hilde Verbeek, S. M. G. Zwakhalen, J. M. G. A. Schols, G. I. J. M. Kempen, J. P. H. Hamers

**Affiliations:** Living Lab in Ageing and Long-Term Care, Department of Health Services Research, CAPHRI, Faculty of Health, Medicine and Life Sciences, Maastricht University, Maastricht, the Netherlands

**Keywords:** Home care, nursing homes, research partnership, knowledge infrastructure

## Abstract

There is a strong need in long-term care for scientific research, so older people and their families, health care professionals, policy makers, and educators can benefit from new advancements and best available evidence in every day care practice. This paper presents the model of a sustainable and successful interdisciplinary collaboration between scientists, care providers and educators in long-term care: the “Living Lab in Ageing and Long-Term Care” by Maastricht University in the Netherlands. Its mission is to contribute with scientific research to improving i) quality of life of older people and their families; ii) quality of care and iii) quality of work of those working in long-term care. Key working mechanisms are the Linking Pins and interdisciplinary partnership using a team science approach, with great scientific and societal impact. A blueprint for the model is discussed, describing its business model and challenges in getting the model operational and sustainable are discussed.

Demand for long-term care is rising, because of the aging of the population and the increase in chronic and degenerative diseases (1). Both community and institutional care services are facing challenges, not only caused by aggregated care needs but also through technological and related health care innovations. At the same time, the number of well-trained staff is decreasing, partly due to a negative image of care provision within the field of geriatrics (2). Innovations that address the changing needs and demands of the future are highly warranted and there is a strong need to develop and implement evidence-based practice and technology (3). Unfortunately, it may take on average up to 17 years before scientific knowledge is adopted in daily practice (4), and there is not a strong tradition of scientific research in long-term care. The result is that older people and their families, health care professionals, policy makers, and educators do not benefit sufficiently from new advancements and best available evidence.

This paper presents a model for a sustainable and successful interdisciplinary collaboration between scientists, care providers, and educators in long-term care: the “Living Lab in Ageing and Long-Term Care” by Maastricht University in the Netherlands. For over 20 years this structural collaboration has served as an infrastructure that drives scientific research in long-term care in co-creation with end-users, including older people and their relatives, health care professionals, policy makers and educators. From 2018, the model has been granted with structural co-funding by the Dutch government of €1 million annually, as a standard for building a substantial knowledge infrastructure in long-term care in the Netherlands. The Living Lab receives an increasing number of invitations to present the model in a variety of countries all over the world, including Europe, the United States, China and Australia.

The purpose of this paper is to describe the aim of the Living Lab and explain the key working mechanisms of the interdisciplinary collaboration, highlighting the scientific and societal impact. We will provide a blueprint for the model, describe its business model, and discuss challenges in getting the model operational and sustainable.

## Living Lab in Ageing and Long-Term Care

The Living Lab in Ageing and Long-Term Care was founded in 1998 by the structural collaboration between a nursing home and Maastricht University. The impetus was a desire to embed scientific research within long-term care for older people (5). In 2000, the collaboration was expanded with two other long-term care organizations and Zuyd University of Applied Science (responsible for training of bachelor educated professionals, e.g. nurses, physiotherapists, social workers). Today, the Living Lab is a sustainable and successful partnership of Maastricht University with seven long-term care providers, Zuyd University of Applied Sciences and two vocational training institutes (responsible for training of e.g. nursing assistants).

The Living Lab is not a physical space, but a network in the southern part of the Netherlands in which researchers collaborate through continuous discussion with end-users such as older persons and their families, client representatives, professionals, health care directors, policy makers and teachers. It covers approximately 110 long-term care facilities (e.g. nursing homes, assisted and group living facilities) as well as professional home care, and includes about 30,000 clients and more than 15,000 staff (see Box 1).

Box 1 Facts & Figures 2019 Within the Living Lab on Ageing and Long-Term Care there are approximately:

- 20 Linking Pins
- 20 client representatives
- 50 scientific researchers
- 50 staff members from partner organizations
- 5 research assistants, secretary and communication staff
- 500 staff from care providers annually involved in specific projects

The mission of the Living Lab is to contribute through scientific research to improving (i) quality of life for older people and their families; (ii) quality of care, and (iii) quality of work for those employed in long-term care. The Living Lab targets long-term care service delivery for older people, including home care, institutional care, palliative care and geriatric rehabilitation. The Living Lab collaborates closely on research projects with relevant stakeholders from different domains, depending on the objectives, such as primary care, hospital, mental health services, local municipalities and businesses. Older people and their relatives and representatives have a central role within the Living Lab. Clients and/or their representatives participate directly in each research project, either as a member of the project group or advisors/consultants throughout the project. On a regular basis (approximately three times a year), a group of client representatives from the seven long-term care organizations advise, reflect on results, and help set future directions as strategic partners. In addition, the Living Lab has a partnership with the Dutch Council for Older People, who advise the Minister of Health, Welfare and Sports directly.

**Figure 1:**
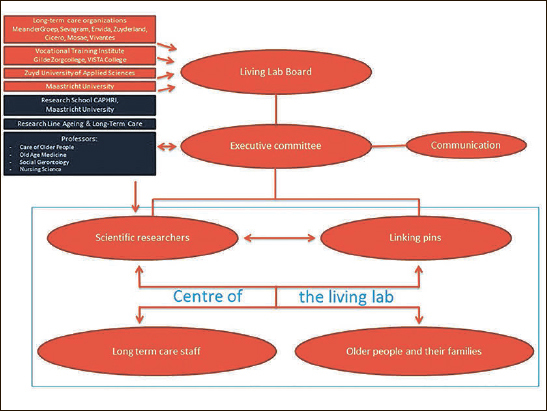
Organizational structure of the Living Lab in Ageing and Long-Term Care

The organizational structure of the Living Lab is presented in Figure 1. The Living Lab model has two vital characteristics (5), that form the center of all activities. The first is the use of Linking Pins, and the second is the use of an interdisciplinary partnership that is based on a team science approach.

The strength behind the Living Lab is the Linking Pins, who hold joint appointments at the University and at long-term care organizations or vocational training institutes. There are two types of Linking Pin roles within each partner organization — scientific and practice-based — that work as couples in reciprocity with each other. Together the Linking Pins build an infrastructure to stimulate scientific research within long-term care. Scientific Linking Pins are senior researchers who work for one or two days a week in one of the partner organizations. Their role is to coordinate scientific research and teaching activities, lead a multidisciplinary working group of long-term care professionals, and together with staff and older people assist in identifying problems in practice that need further investigation. The practice-based Linking Pin is an employee of the partner organization who works for one day a week on scientific research and facilitation of scientific knowledge and innovations in practice. The Linking Pin couple translate the projects to the individual partner organization.

The second key characteristic of the Living Lab is an interdisciplinary partnership using a team science approach. Collaborating partners include older people and their families, and a wide variety of health care professionals, managers and directors, policy makers, service designers, companies, educators and funding agencies (e.g. research funders, health care insurers, government). Interdisciplinary groups work together on specific research projects. Partners decide in which projects they want to participate and also organizations from outside the Living Lab can participate in the research projects as well. Scientific disciplines represented in the Living Lab are nursing science, old age medicine, social gerontology, psychology, physiotherapy, occupational therapy, health sciences and customer-centric service design.

Each partner organization is represented by their CEO at the general board meeting of the Living Lab, which makes decisions regarding overall themes, spearheads and implications of results. The executive committee of the board is responsible for the day-to-day management of the Living Lab. This includes providing supervision to Linking Pins and researchers, discussing general Living Lab results and projects with stakeholder groups both internally (e.g. meetings with communication officers, policy officers, and client representatives of the partner organizations) and externally (e.g. with the Ministry of Health, Welfare and Sports, other academic networks and regional stakeholders). In addition to direct partners, other long-term care facilities and stakeholders participate depending on the objectives of specific research projects.

## The Business Model of the Living Lab

Two aspects of the Living Lab model require funding: a) infrastructure, and b) research. Partner organizations are the principal funders with respect to infrastructure, while grants provide the primary financial support for research.

## Infrastructure

Long-term care organizations fund infrastructure by paying for the scientific Linking Pins. Formally, the long-term care organizations have no allocated budget for scientific research. They account for these amounts through the impact research projects have on innovation and improvement of practice for clients, their relatives and staff. In addition, all partner organizations contribute in kind, through allocating resources enabling staff to work on Living Lab projects (including time and training). Maastricht University finances infrastructure by paying for the coordination of the Living Lab, including supportive staff and communication. The University also covers the costs of the workplaces of practice-based Linking Pins at the university. As this model has proven its sustainability over the last 20 years, the Dutch Ministry of Health, Welfare and Sports has decided in 2018 to financially support the infrastructure investments with €1 million, strengthening the Living Lab’s infrastructure and power.

## Research

The Living Lab model requires funding for scientific research, which is primarily provided by externally acquired competitive grants (e.g., European grants such as within Horizon 2020 calls, and national grants from the Netherlands Organization for Health Research and Development). Additionally, partner organizations sometimes fund or co-fund specific projects that they perceive as high priority research, and for which no external funding is available. Examples include research projects on the development of an assessment instrument of quality of care from the client perspective (6), involuntary treatment of people with cognitive impairment living at home (7). The Living Lab acquires on average €1,5 million external research funds per year. Its research results annually in approximately 3 PhD theses, 45 international peer-reviewed scientific publications and 50 abstracts and keynotes for conferences.

## Scientific Research and Education

**Figure 2:**
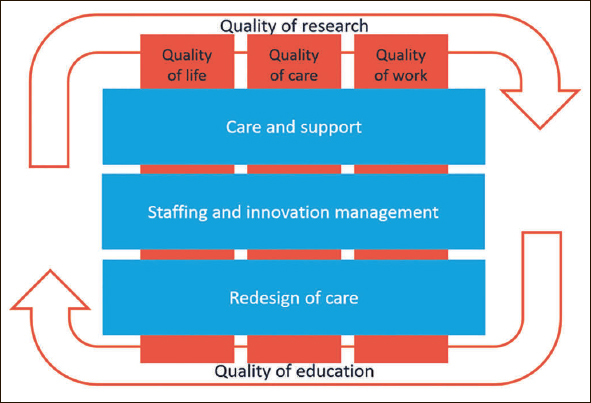
Research Lines Maastricht Living Lab Ageing and Long-Term Care

The Living Lab partnership uses scientific research to address problems identified by people who live and work in long-term care. The continues dialogue between researchers and all end-users has identified three main research themes have been identified for the Living Lab (Figure 2):

- Care and support improving the daily life of older people and their families;
- Staffing and innovation management;
- Redesign in long-term care practice;

The first research theme focuses on improving the daily life of older people and their families through improvements in support, delivery of care services and treatment. This research takes place at the micro level of care service delivery, that is, in the direct care relationship with older people, their families and professionals. Examples of these research projects include the National Prevalence Measurement of Quality of Care (https://nl.lpz-um.eu/nl), improving palliative care for people with dementia (8) or developing diagnostic techniques for heart failure in nursing homes (9).

Scientific knowledge on how best to equip professionals in doing their job is imperative to improve support and care service delivery. The second research theme, therefore, focuses on staffing and innovation management in long-term care. Example projects include optimal skills-mix, team culture and organizational climate (10), development of a decision-support application for community nurses and case managers in dementia care (11), all aimed at evidence-based innovation of care service delivery and support.

Radical alterations are sometimes necessary to change current long-term care practice. The third research theme includes studies on the impact of redesign in long-term care practice, focusing on how care service delivery can be improved in order to better meet the needs and wishes of older people and their families. Example projects include evaluation of green care farms as an alternative to traditional nursing homes (12), development and evaluation of a geriatric rehabilitation care pathway (13) or population-based financing in home care (14).

The Living Lab intends to improve the quality of scientific research. This includes the development of adequate assessment instruments for important outcome measures in long-term care, such as the identification of pain in people with dementia (15) or assessment of daily life in nursing homes (16). Scientific research conducted within the Living Lab uses a variety of research methodologies and paradigms, including pragmatic trials and action-based research approaches.

The Living Lab also aims to improve the quality of education. It is important to encourage enthusiasm for young people to choose a professional career in the field of geriatrics and gerontology and to build an infrastructure of health care innovators for long-term care. Up-to-date scientific knowledge is implemented in educational programs for future students on all levels: from vocational training to bachelor’s and master’s degree programs. The Living Lab also enables students to work within innovative research projects. To illustrate, the Living Lab developed in 2018 a new bachelor’s program for nurses led by Zuyd University of Applied Sciences. Unique to the program is that students are employed by one long-term care organization during the study program but receive training and education in a learning network across the Living Lab partner organizations. Themes such as leadership, palliative care, innovation and technology are being taught directly by professionals of the long-term care organizations and researchers working within the Living Lab. Educators from Zuyd University of Applied Sciences and the vocational training institutes provide innovative didactic techniques and support. Furthermore, we have integrated Virtual Reality techniques have been integrated into university courses for bachelor’s degree students to learn about innovations in home care (17). At the same time, this educational module is used within the partner long-term care organizations to train home care staff in principles of reablement (18).

## Impact of the Living Lab

Over the past 20 years, the Living Lab on Ageing and Long-Term Care has had a highly significant scientific and societal impact. For direct partners, the Living Lab has increased enthusiasm, (scientific) awareness and expertise of staff through structural participation in projects, education and sharing of experiences at annual symposia. The Living Lab provides a safe, non-competitive environment, in which there is space for people to engage in a dialogue and to experiment. This creates a learning network of researchers, older people and their families, and professionals to encourage innovation in long-term care practice. This goes beyond the borders of the Living Lab, as work visits and exchanges have been organized with other groups of long-term care researchers, such as in the United Kingdom (My Home Life, Leeds NICHE).

Scientifically, the Living Lab has enabled the development of new knowledge in a variety of areas within long-term care. In its 20 years, the research has led to the publication of over 750 peer-reviewed scientific papers in international journals and 25 PhD theses. The partnership is attractive for external funding agencies, which adding to its earning power. Tools and interventions have been developed to support professionals in long-term care (9, 11, 13, 15). The Living Lab has developed knowledge on innovative forms of long-term care, such as small-scale homelike facilities (12, 19) and function-focused care at home (18).

The Living Lab research has had an impact on national policy development through changes in legislation (e.g. reduction of restraints and involuntary treatment) and changes in policy (e.g. relationship between staffing and quality of care in nursing homes). Furthermore, it has taken a role in the societal debate on long-term care, by organizing public meetings for citizens and by developing factsheets for lay audience and video messaging. Its meeting in December 2018, where the Living Lab celebrated its 20th Anniversary, attracted almost 1100 people, including older citizens, long-term care staff and students.

The Living Lab provides an attractive environment for young students to gain enthusiasm for working in long-term care. Students can participate in research projects and can take on new positions and leadership roles. Furthermore, the Living Lab assist in training people to become health care innovators within long-term care.

## Implications for practice and research

Sharing knowledge about the Living Lab may facilitate others in setting up a similar model for creating a partnership between research and long-term care practice. It is important to start small with a selected group of motivated partners. Intrinsic motivational commitment from these parties is necessary for innovation, change and improvement. The establishment of the partnership should require no extra funding, only cash and in-kind funding from the partners to set up the collaboration. Starting small will enhance the success rate of selected projects and increase the belief that such translational model may really work. This can increase enthusiasm from other interested partners and is necessary to develop a mutual trusting relationship among partners, which is a key element for success. The network can then gradually be increased to enable and jointly develop the mission and vision of the Living Lab and to train researchers and Linking Pins, building a full infrastructure within the individual partner organizations.

Expectations from all partners should be managed carefully and should not be set too high, especially at the beginning. No major results can be expected immediately after the start. For example, a partnership will not solve policy issues or practical problems in daily care practice for the whole organization right away. The driving force behind the Living Lab is the reciprocity and trust among partners. To create a meaningful collaboration, it is necessary that both partners have a vision on collaboration and experience benefit to create a win-win perspective. This requires commitment of partners, efforts in establishing a trusting relationship and confidence in each other. Sympathy for each other is crucial in this process. This accounts for sympathy between academia, practice, and education but also across the various disciplines working within the Living Lab. It takes an effort in trying to understand each other’s viewpoint. However, improving the quality of life for older people and their families, quality of care and quality of work for staff in long-term care, unites the partners. Equity and finding tailor-made solutions based on scientific research for individual partners is part of the success of the Living Lab.

## Future Directions

Maastricht University’s Living Lab on Ageing and Long-Term Care may be viewed as a best-practice example, and its approach has been adopted by other (inter)national groups. The Nurturing Innovation in Care Home Excellence partnership was established in 2018 in Leeds (United Kingdom) based on the Maastricht’s Living Lab blueprint. Furthermore, Maastricht’s Living Lab was a model partner in the Horizon2020 Twining Project ALTHOUR (Assisted Living Technology for the Health Tourism sector), in which its’ structure and research was a prototype example of a Living Lab structure to prepare for the set-up of a ‘Health Tourism Living Lab’ in the Lisbon area (Portugal) by the University of Lisbon. After the start of the Maastricht Living Lab, there are five other academic networks established in long-term care in The Netherlands, situated in Amsterdam, Leiden, Nijmegen, Tilburg and Groningen. They all act from the same principle, aiming to embed scientific research into daily LTC-practice. To some extent, differences exist in organization, interdisciplinarity and research focus. However, they have all adopted the linking-pin model as developed by Maastricht. Nationally, the 6 networks meet regularly to exchange experiences and good practices and also to discuss strategies to further implement evidence based practice in long-term care, to ultimately cover the total LTC-sector in the country.

There is no fixed scheme to set up a partnership between academia and long-term care providers and associated organizations, as one size does not fit all. Both within and between countries, organizational culture, structure, and financing of long-term care and research differ, affecting the organizational structure of a partnership. However, in our opinion, the interdisciplinary character and Linking Pins are responsible for the success of the Living Lab on Ageing and Long-Term Care over the past 20 years. This creates a sustainable learning network across organizations, which is highly recommended to create a research culture in long-term care (20).
